# Supplementary material for: Genomewide Analysis and Biological Characterization of Cathelicidins with Potent Antimicrobial Activity and Low Cytotoxicity from Three Bat Species
Source: Antibiotics (Basel). 2022 Jul 22;11(8):989. doi: 10.3390/antibiotics11080989 (PMC9330922; doi:10.3390/antibiotics11080989)
Supplement: Supplementary file 1 [file antibiotics-11-00989-s001.zip › antibiotics-1821787-supplementary Figure S1.pdf]

Supplementary Material

Peptides	Amino acid properties of three bat cathelicidins																			
	1	2	3	4	5	6	7	8	9	10	11	12	13	14	15	16	17	18	19	20
HA-CATH	I	L	G	R	L	R	D	L	L	R	R	G	G	R	K	I	G	Q	G	L
	A/N	A/N	A/N	P	A/N	P	N	A/N	A/N	P	P	A/N	A/N	P	P	A/N	A/N	U/P	A/N	A/N
ML-CATH	L	N	P	L	I	K	A	G	I	F	I	L	K	H	R	R	P	I	G	R
	A/N	U/P	U/P	A/N	A/N	P	A/N	A/N	A/N	A	A/N	A/N	P	P	P	P	U/P	A/N	A/N	P
PD-CATH	I	L	G	P	A	L	R	I	G	G	R	I	A	G	R	I	A	G	K	L
	A/N	A/N	A/N	U/P	A/N	A/N	P	A/N	A/N	A/N	P	A/N	A/N	A/N	P	A/N	A/N	A/N	P	A/N

Peptides	Amino acid properties of three bat cathelicidins																			
	21	22	23	24	25	26	27	28	29	30	31	32	33	34	35	36	37	38	39	40
HA-CATH	E	R	I	G	Q	R	I	Q	G	F	F	S	N	R	E	P	M	E	E	S
	N	P	A/N	A/N	U/P	P	A/N	U/P	A/N	A	A	U/P	U/P	P	N	U/P	A/N	N	N	U/P
ML-CATH	G	I	E	I	T	G	R	G	I	K	K	F	F	S	K					
	A/N	A/N	N	A/N	U/P	A/N	P	A/N	A/N	P	P	A	A	U/P	P					
PD-CATH	I	G	D	A	I	N	R	H	R	E	R	N	R	Q	R	R	G			
	A/N	A/N	N	A/N	A/N	U/P	P	P	P	N	P	U/P	P	U/P	P	P	A/N			

U/P (Uncharged, Polar)   A/N (Aliphatic, Nonpolar)   A (Aromatic, Nonpolar)   P (Positively charged)   N (Negatively charged)

Figure S1: Comparison of the amino acid sequences and properties of the three bat cathelicidins evaluated in this study. Amino acid sequences of cathelicidins and their chemical properties are shown in different colors corresponding to each amino acid. Their chemical properties are defined at the bottom. Numbers at the top indicate the positions of amino acids.
